# Physical Activity, Sedentary Behaviour and Mental Health in University Students: A Compositional Isotemporal Substitution Analysis

**DOI:** 10.1155/da/4091641

**Published:** 2026-09-03

**Authors:** Flavia Pennisi, Antonio Pinto, Marco Colizzi, Carlo Signorelli, Andrea Cozza, Vincenzo Baldo, Vincenza Gianfredi

**Affiliations:** ^1^ Faculty of Medicine, University Vita-Salute San Raffaele, Milan, Italy, unisr.it; ^2^ PhD National Program in One Health Approaches to Infectious Diseases and Life Science Research Department of Public Health, Experimental and Forensic Medicine, University of Pavia, Pavia, Italy, unipv.eu; ^3^ Unit of Psychiatry, Department of Medicine (DMED), University of Udine, Udine, Italy, uniud.it; ^4^ Department of Psychosis Studies, Institute of Psychiatry, Psychology and Neuroscience, King’s College London, London, UK, kcl.ac.uk; ^5^ Department of Cardiac Thoracic Vascular Sciences and Public Health, University of Padua, Padua 35128, Veneto, Italy, unipd.it

**Keywords:** compositional data analysis, depressive symptoms, eating disorder, isotemporal substitution, physical activity, sedentary behaviour

## Abstract

This cross‐sectional study examined the associations between waking movement behaviour composition and depressive symptoms, self‐rated health (SRH) and eating‐disorder (ED)‐related symptomatology (SCOFF) in university students using compositional data analysis (CoDA) and isotemporal substitution modelling. Data were collected through an anonymous web‐based survey among students enrolled at the University of Milan, Italy. The analytical sample included 2636 participants. International Physical Activity Questionnaire (IPAQ) was used to measure daily time spent in moderate‐to‐vigorous physical activity (MVPA), light physical activity (LPA), and sedentary behaviour (SB) and analysed as a three‐part composition. Outcomes included depressive symptoms (Patient Health Questionnaire‐9 [PHQ‐9]), SRH and SCOFF score. In adjusted compositional regression models, a higher relative proportion of MVPA was associated with lower depressive symptoms (*β* = −0.39, 95% confidence interval [CI]: −0.53 to −0.24) and higher SRH (*β* = 0.11, 95% CI: 0.08–0.13), whereas a higher relative proportion of SB was associated with higher depressive symptoms (*β* = 0.42, 95% CI: 0.21–0.64) and lower SRH (*β* = −0.11, 95% CI: −0.14 to −0.07). LPA was not significantly associated with outcomes. Isotemporal substitution analyses showed that reallocating 15 min from SB to MVPA was associated with lower PHQ‐9 scores (−0.114, 95% CI: −0.156 to −0.072), higher SRH (0.031, 95% CI: 0.024–0.038) and SCOFF score (*β* = 0.04, 95% CI: 0.00–0.07). These findings suggest that, within the daily movement composition, increasing a greater relative allocation of time to MVPA at the expense of sedentary time is associated with modestly more favourable mental and perceived health in university students.

## 1. Introduction

University students represent a population particularly vulnerable to psychological distress, with depressive symptoms, impaired self‐perceived health and disordered eating behaviours emerging as relevant public health concerns during the transition to adulthood [1–5]. At the same time, this life stage is often characterised by unfavourable movement patterns, including insufficient physical activity and prolonged sedentary behaviour (SB), which may contribute to poorer mental and general health outcomes [6, 7]. In Italy, a recent systematic review and meta‐analysis reported pooled prevalence estimates of 46% for depressive symptoms, 65% for anxiety symptoms and 85% for significant stress among university students. However, these estimates were characterised by substantial heterogeneity and were partly influenced by studies conducted during the COVID‐19 pandemic [8].

Growing evidence indicates that movement behaviours should not be examined in isolation, as time spent in one behaviour necessarily displaces time spent in another within the finite framework of waking hours [9, 10]. Accordingly, compositional data analysis (CoDA) has been increasingly advocated as the appropriate methodological approach to study the joint and codependent nature of daily behaviours [11, 12]. This framework may offer additional insights compared with conventional models, particularly when combined with isotemporal substitution analyses that model the changes in health outcomes associated with hypothetical reallocations of time between behaviours [13, 14]. Although the associations of physical activity and SB with mental health have been widely investigated [15–18], evidence remains limited in university populations when movement behaviours are analysed as an integrated composition. Moreover, less is known about how different reallocations of time between moderate‐to‐vigorous physical activity (MVPA), light physical activity (LPA) and SB relate not only to depressive symptoms but also to self‐rated health (SRH) and eating‐disorder (ED)‐related symptomatology. Therefore, the present study aimed to examine the associations between waking movement behaviour composition and these health outcomes in a large sample of university students using CoDA and isotemporal substitution modelling.

## 2. Materials and Methods

### 2.1. Study Design and Setting

The current study was conducted as an observational cross‐sectional survey among students enrolled at the University of Milan (Italy). The survey targeted students registered in undergraduate, graduate or postgraduate programs during the study period. Data were collected through an anonymous web‐based questionnaire developed using Microsoft Forms.

The survey link was disseminated through the University’s official mailing system and could be accessed only after authentication with institutional email credentials. Authentication was used solely to verify student eligibility and prevent unauthorised or multiple submissions. Institutional email addresses and authentication credentials were not recorded in the questionnaire dataset and were not linked to the participants’ responses, thereby preserving anonymity. This access restriction ensured that participation was limited to actively enrolled students. Participation was voluntary. Electronic informed consent was required before respondents could proceed with the questionnaire. To minimise incomplete responses, all questionnaire items were configured as mandatory fields. A detailed description of the study protocol has been published previously [19].

### 2.2. Participants

All students aged 18 years or older enrolled at the University of Milan during the study period were eligible for participation.

### 2.3. Movement Behaviour Assessment (Exposure Variable)

Movement behaviours were assessed using self‐reported measures. Physical activity was measured with the short form of the International Physical Activity Questionnaire (IPAQ‐SF), which captures the frequency and duration of vigorous‐intensity activity, moderate‐intensity activity and walking performed for at least 10 consecutive minutes during the previous 7 days. Data processing followed the official IPAQ Guidelines for Data Processing and Analysis (revised in November 2005) [20].

The average daily time spent walking and performing moderate‐ and vigorous‐intensity physical activity was estimated by multiplying the number of reported days by the average duration (minutes per day) of each activity type. The resulting weekly totals were divided by seven to obtain mean daily durations. Following the IPAQ‐SF data processing guidelines, extreme values exceeding 180 min per day were truncated by imposing an upper limit of 1260 min per week for each physical activity domain, including vigorous‐intensity activity, moderate‐intensity activity and walking.

Mean daily minutes of moderate‐ and vigorous‐intensity activity were subsequently summed to derive total MVPA. Time spent walking was used as an indicator of LPA. Both MVPA and LPA were analysed as continuous variables expressed in minutes per day.

SB was assessed as self‐reported average daily sedentary time and was analysed as a continuous variable expressed in minutes per day. For the compositional analyses, daily time spent in MVPA, LPA and SB was treated as a three‐part movement behaviour composition representing waking time.

### 2.4. Health Outcomes

Depressive symptoms were assessed using the Italian validated version of the Patient Health Questionnaire‐9 (PHQ‐9), a widely used screening instrument for depressive symptom severity in epidemiological research [21, 22]. The PHQ‐9 consists of nine items assessing the frequency of depressive symptoms experienced during the previous 2 weeks. Each item is scored on a four‐point Likert scale ranging from 0 (‘not at all’) to 3 (‘nearly every day’), yielding a total score ranging from 0 to 27. The PHQ‐9 total score was analysed as a continuous variable, with higher scores indicating greater depressive symptom severity.

SRH was evaluated through a single question (‘In general, how would you rate your health?’), with response options ranging from excellent to poor [23, 24]. Responses were coded on a 5‐point scale and analysed as a continuous variable, with higher scores indicating better perceived health. SRH is widely recognised as a reliable and validated predictor of subsequent health outcomes [25, 26].

The Italian version of the SCOFF questionnaire, a validated five‐item screening instrument, was used to screen participants for ED‐related symptomatology. Each item requires a dichotomous response (‘Yes’ = 1 and ‘No’ = 0), generating a total score ranging from 0 to 5 [27, 28]. The questionnaire addresses key symptoms associated with ED, including self‐induced vomiting after overeating (Sick), loss of control over food intake (Control), recent weight loss of at least 6 kg within 3 months (One stone), perception of being overweight despite others’ reassurance (Fat) and persistent thoughts about food (Food). Higher total scores reflect a greater likelihood of ED‐related symptomatology. The Italian validated version demonstrates acceptable internal consistency (Cronbach’s *α* = 0.64) [24].

### 2.5. Covariates

Gender, age category and educational level were included as covariates to account for potential confounding in the regression models. Gender was categorised as women, men or ‘prefer not to say’. The educational level was classified as high school diploma, bachelor’s degree or master’s degree and above.

### 2.6. Sample Size Estimation

The sample size was estimated assuming a 95% confidence level and a margin of error of 5%. The reference population corresponded to all students enrolled at the University of Milan during the 2021/2022 academic year (*N* = 60,988) [29].

Because reliable prevalence estimates of depressive symptoms among Italian university students were not available during study planning, a conservative expected prevalence of 50% was used. Under these assumptions, the minimum required sample size was estimated to be 382 participants.

### 2.7. Bias and Data Quality Procedures

Several procedures were implemented to ensure data quality and reduce potential sources of bias. The online questionnaire included mandatory fields to limit the number of missing responses. Before statistical analyses, the dataset was screened for duplicate entries, internal inconsistencies and implausible values.

**Figure 1:**
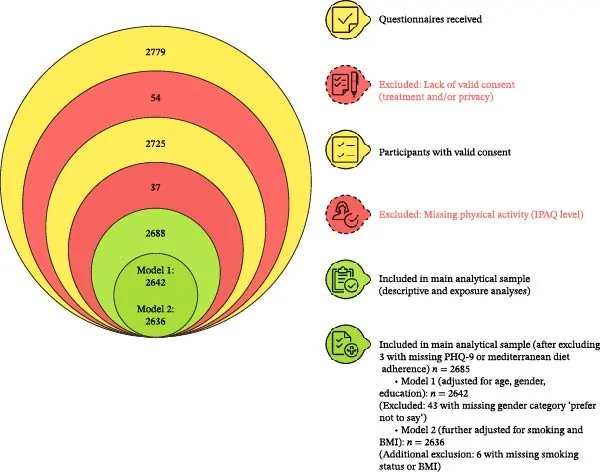
Flow diagram of participant selection and final analytical samples.

The sample selection process is illustrated in Figure 1. A total of 2779 questionnaires were initially received. Responses without valid informed consent (missing acceptance of treatment and/or privacy conditions) were excluded (*n* = 54), leaving 2725 participants with valid consent. Participants with missing information (due to a technical problem in the Microsoft Form) on physical activity derived from the IPAQ questionnaire were further excluded (*n* = 37), resulting in 2688 individuals included in the main analytical sample.

For outcome analyses, participants with missing information on PHQ‐9 were additionally excluded (*n* = 3), yielding a final analytical sample of 2685 participants. In multivariable regression analyses, further exclusions were applied due to missing covariate data: Model 1 included 2642 participants after excluding individuals with the missing gender category (“prefer not to say”), and Model 2 included 2636 participants after an additional exclusion of participants with the missing smoking status or BMI.

### 2.8. Ethical Considerations

The study was conducted in accordance with the principles of the Declaration of Helsinki and complied with national regulations regarding the protection of personal data. Ethical approval was obtained from the Ethics Committee of the University of Milan (Approval ID: 71.23). Participation was voluntary, and electronic informed consent was obtained from all respondents before participation. The questionnaire was fully anonymous and did not collect personal identifiers such as names, student identification numbers, or IP addresses. Participants were informed about the objectives of the study, confidentiality safeguards, and their right to withdraw before submitting the questionnaire. Data were stored in password‐protected files accessible only to the research team.

### 2.9. Reporting Standards

The reporting of this study follows the Strengthening the Reporting of Observational Studies in Epidemiology (STROBE) guidelines for cross‐sectional studies [30] (Table S1).

### 2.10. Statistical Analysis

Descriptive statistics were computed to summarise the characteristics of the study sample and the distribution of movement behaviours. Continuous variables were expressed as means and standard deviations. Pearson correlation coefficients, together with their 95% confidence intervals (95% CIs), were calculated to assess bivariate associations between movement behaviours and health outcomes.

Because daily movement behaviours represent mutually exclusive components of a finite time budget, they were analysed within a CoDA framework. In compositional data, total time is constrained, meaning that an increase in time allocated to one behaviour necessarily implies a decrease in time allocated to at least one other behaviour. Consequently, movement behaviours must be interpreted on a relative rather than absolute scale.

Daily time spent in MVPA, LPA and SB was treated as a behavioural composition representing waking time. To account for the relative structure of compositional data and permit their inclusion in standard regression models, the composition was transformed using isometric log‐ratio (ilr) coordinates. Two ilr coordinates were derived using a pivot coordinate approach, allowing each behaviour to be expressed relative to the remaining components of the composition.

Separate linear regression models were fitted to examine the associations between movement behaviour composition and the study outcomes, including depressive symptoms (PHQ‐9 score), SRH and SCOFF score. In each model, the ilr coordinates were entered as predictors. All models were adjusted for gender, age category and education level.

To facilitate the interpretation of the compositional regression results, compositional isotemporal substitution analyses were conducted. This approach estimates the expected change in the outcomes associated with reallocating time between movement behaviours while keeping total daily time constant. The mean behavioural composition of the study sample was used as the reference composition.

Predicted changes in each outcome were estimated by reallocating time between behaviours in 5‐min increments up to 60 min. For each reallocation scenario, the corresponding variation in the ilr coordinates was calculated and used to derive the predicted change in the outcome based on the fitted regression models. Predicted estimates and corresponding 95% CIs were obtained using linear combinations of the regression coefficients.

Results of the isotemporal substitution analyses were presented both in tabular form for a 15‐min reallocation and graphically to illustrate the predicted changes in PHQ‐9, SRH and SCOFF scores associated with increasing or decreasing time allocated to each movement behaviour.

All statistical tests were two‐sided and statistical significance was set at *p*  < 0.05. All statistical analyses were performed using Stata 17 (StataCorp LLC, College Station, TX, USA).

## 3. Results

### 3.1. Sample Characteristics and Correlations

The analytical sample included 2636 participants. In the overall sample, 70.6% were women, 27.8% were men and 1.6% preferred not to report their gender. Most participants were aged 18–23 years (52.5%), followed by 24–29 years (31.7%) and ≥30 years (15.7%). Regarding educational level, 52.6% reported a high school diploma, 25.7% a bachelor’s degree and 21.7% a master’s degree or higher.

Mean daily time spent in movement behaviours was 40.84 min for MVPA, 57.73 min for LPA and 320.75 min for SB. The mean PHQ‐9 score was 7.53 (SD = 5.13), and the mean SCOFF score was 1.92 (SD = 1.27). The mean SRH score was 3.33 (SD = 0.85) on a 5‐point scale (Table 1).

**Table 1:** Descriptive statistics and Pearson correlations between movement behaviours and health outcomes (*n* = 2636).

#	Variabile	Mean	SD	1	2	3	4	5	6
1	MVPA (min/day)	40.84	46.33	—	—	—	—	—	—
2	LPA (min/day)	57.73	51.45	0.221 [0.184; 0.257] ^∗∗∗^	—	—	—	—	—
3	SB (min/day)	320.75	138.89	−0.103 [−0.141; −0.065] ^∗∗∗^	−0.052 [−0.090; −0.014] ^∗∗^	—	—	—	—
4	PHQ‐9 total	7.53	5.13	−0.045 [−0.083; −0.006] ^∗^	0.033 [−0.006; 0.071]	0.097 [0.058; 0.135] ^∗∗∗^	—	—	—
5	SRH	3.33	0.85	0.160 [0.123; 0.198] ^∗∗∗^	0.007 [−0.031; 0.046]	−0.028 [−0.067; 0.010]	−0.332 [−0.366; −0.298] ^∗∗∗^	—	—
6	SCOFF score	1.92	1.27	0.027 [−0.012; 0.065]	0.046 [0.008; 0.084] ^∗^	−0.039 [−0.078; −0.001] ^∗^	0.399 [0.366; 0.431] ^∗∗∗^	−0.193 [−0.229; −0.155] ^∗∗∗^	—

*Note:* Values are presented as means and standard deviations for continuous variables. Pearson correlation coefficients are reported with 95% confidence intervals in brackets, calculated using Fisher’s z transformation. SRH was coded 1–5 (higher scores indicate better health). SCOFF scores range 0–5. PHQ‐9 was treated as a continuous variable.

Abbreviations: LPA, light physical activity; MVPA, moderate‐to‐vigorous physical activity; SB, sedentary behaviour.

^∗^ *p*  < 0.05.

^∗∗^ *p*  < 0.01.

^∗∗∗^ *p*  < 0.001.

Pearson correlations between study variables are presented in Table 1. MVPA showed a small inverse correlation with depressive symptoms (*r* = −0.045, 95% CI: −0.083 to −0.006) and a positive correlation with SRH (*r* = 0.160, 95% CI: 0.123–0.198). SB was positively correlated with PHQ‐9 scores (*r* = 0.097, 95% CI: 0.058–0.135). Associations between LPA and psychological outcomes were small and not statistically significant, with the exception of the SCOFF score (*r* = 0.046, 95% CI: 0.008–0.084).

### 3.2. Associations Between Movement Behaviour Composition and Health Outcomes

Associations between movement behaviour composition and health outcomes are shown in Table 2. In fully adjusted compositional regression models (Model 2), the first pivot coordinate for MVPA was inversely associated with depressive symptoms (*β* = −0.39, 95% CI: −0.53 to −0.24) and positively associated with SRH (*β* = 0.11, 95% CI: 0.08–0.13). A small positive association was observed between MVPA and the SCOFF score (*β* = 0.04, 95% CI: 0.00–0.07).

**Table 2:** Regression coefficients associated with the first pivot coordinates for the association between movement behaviours and health outcomes.

First ilr coordinate	Model	Depressive symptoms (PHQ‐9) *β* (95% CI)	Self‐rated health (SRH) *β* (95% CI)	SCOFF score *β* (95% CI)
MVPA	Model 1	−0.38 [−0.53, −0.24] ^∗∗∗^	0.11 [0.08, 0.13] ^∗∗∗^	0.04 [0.00, 0.07] ^∗^
	Model 2	−0.39 [−0.53, −0.24] ^∗∗∗^	0.11 [0.08, 0.13] ^∗∗∗^	0.04 [0.00, 0.07] ^∗^
LPA	Model 1	−0.03 [−0.24, 0.18]	0.00 [−0.03, 0.03]	0.03 [−0.02, 0.08]
	Model 2	−0.04 [−0.24, 0.17]	0.00 [−0.04, 0.03]	0.03 [−0.02, 0.08]
Sedentary behaviour	Model 1	0.41 [0.20, 0.63] ^∗∗∗^	−0.11 [−0.14, −0.07] ^∗∗∗^	−0.07 [−0.12, −0.01] ^∗^
	Model 2	0.42 [0.21, 0.64] ^∗∗∗^	−0.11 [−0.14, −0.07] ^∗∗∗^	−0.07 [−0.12, −0.01] ^∗^

*Note:* Model 1 (*n* = 2642) was adjusted for age group, gender and educational level. Model 2 (*n* = 2636) was additionally adjusted for smoking status and body mass index (BMI).

Abbreviations: CI, confidence interval; ilr, isometric log‐ratio; LPA, light‐intensity physical activity; MVPA, moderate‐to‐vigorous physical activity.

^∗^ *p*  < 0.05.

^∗∗^ *p*  < 0.01.

^∗∗∗^ *p*  < 0.001.

The first pivot coordinate for SB was positively associated with PHQ‐9 scores (*β* = 0.42, 95% CI: 0.21–0.64) and inversely associated with SRH (*β* = −0.11, 95% CI: −0.14 to −0.07). A small inverse association was also observed between SB and SCOFF score (*β* = −0.07, 95% CI: −0.12 to −0.01). No statistically significant associations were observed for the pivot coordinate representing LPA.

### 3.3. Isotemporal Substitution Analyses

Predicted changes in health outcomes associated with reallocating 15 min between movement behaviours are reported in Table 3.

**Table 3:** Predicted change in health outcomes associated with the reallocation of 15 min between movement behaviours (*n* = 2642).

From\to	MVPA	LPA	Sedentary behaviour
(A) Depressive symptoms (PHQ‐9)			
MVPA	–	0.138 [0.062, 0.214]	0.159 [0.099, 0.218]
LPA	−0.091 [−0.162, −0.019]	–	0.023 [−0.034, 0.080]
Sedentary behaviour	−0.114 [−0.156, −0.072]	−0.022 [−0.068, 0.024]	–
(B) Self‐rated health (SRH)			
MVPA	–	−0.039 [−0.052, −0.027]	−0.043 [−0.053, −0.033]
LPA	0.027 [0.015, 0.039]	–	−0.004 [−0.014, 0.006]
Sedentary behaviour	0.031 [0.024, 0.038]	0.004 [−0.003, 0.012]	–
(C) SCOFF score			
MVPA	–	−0.008 [−0.027, 0.011]	−0.016 [−0.031, −0.002]
LPA	0.002 [−0.016, 0.019]	–	−0.010 [−0.025, 0.004]
Sedentary behaviour	0.012 [0.002, 0.023]	0.009 [−0.003, 0.020]	–

For depressive symptoms, reallocating time toward MVPA was associated with lower predicted PHQ‐9 scores. Replacing 15 min of LPA with MVPA was associated with a change of −0.091 points (95% CI: −0.162 to −0.019), while replacing 15 min of SB with MVPA was associated with a change of −0.114 points (95% CI: −0.156 to −0.072). Reallocations away from MVPA toward LPA or SB were associated with higher predicted PHQ‐9 scores. Substitutions between LPA and SB were small and not statistically significant.

For SRH, reallocating time toward MVPA was associated with higher predicted scores. Substituting 15 min of LPA with MVPA corresponded to an increase of 0.027 points (95% CI: 0.015–0.039), whereas replacing 15 min of SB with MVPA corresponded to an increase of 0.031 points (95% CI: 0.024–0.038). Reallocations away from MVPA were associated with lower predicted SRH values.

For the SCOFF score, the estimated changes were smaller. Replacing 15 min of SB with MVPA was associated with an increase of 0.012 points (95% CI: 0.002 to 0.023), while other reallocations produced small and mostly non‐significant changes.

**Figure 2:**
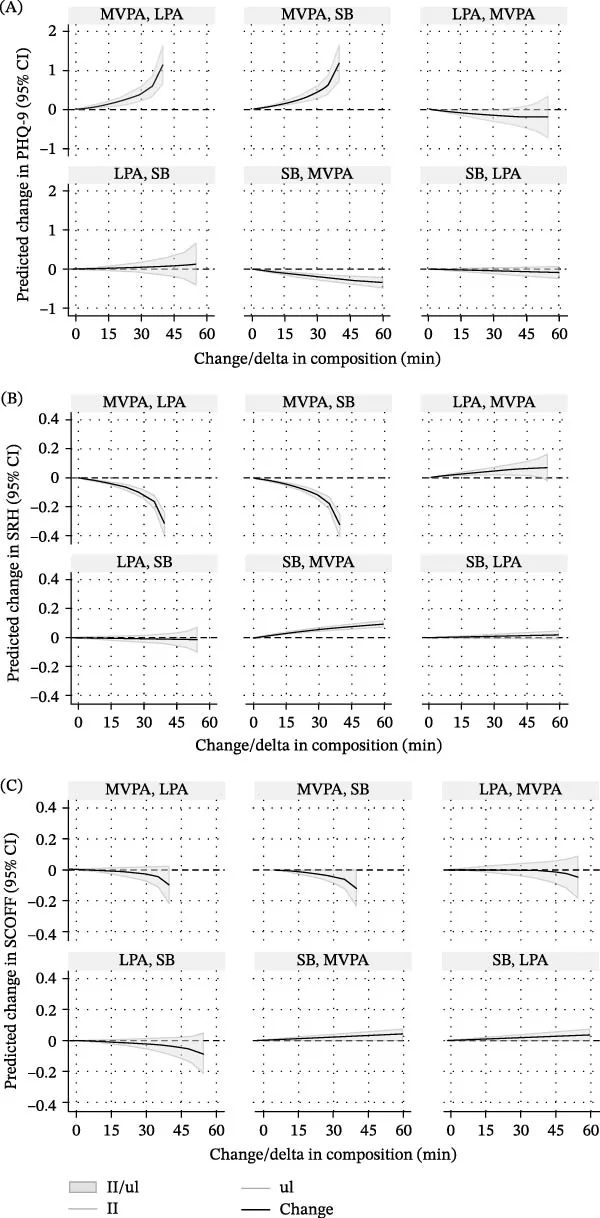
Predicted changes in health outcomes associated with reallocating time between movement behaviours. Predicted changes in (A) depressive symptoms (PHQ‐9), (B) self‐rated health (SRH) and (C) SCOFF score associated with reallocating time between moderate‐to‐vigorous physical activity (MVPA), light physical activity (LPA) and sedentary behaviour (SB). Estimates were derived from compositional isotemporal substitution models using the mean behavioural composition as the reference. The solid line represents the predicted change and the shaded area indicates the 95% confidence interval. Models were adjusted for gender, age category, and educational level.

Figure 2A–C illustrates the predicted changes in health outcomes associated with reallocating time between MVPA, LPA and SB while holding total waking time constant and using the mean behavioural composition as the reference. Reallocations toward MVPA were consistently associated with lower predicted PHQ‐9 scores and higher SRH values, whereas reallocations toward SB showed the opposite pattern. Predicted changes in SCOFF scores were generally smaller in magnitude compared with those observed for PHQ‐9 and SRH.

## 4. Discussion

In this cross‐sectional study of university students, we examined the associations between the daily composition of movement behaviours and several indicators of mental and perceived health using a CoDA framework. Three main findings emerged. First, the relative proportion of time allocated to MVPA was associated with more favourable health profiles, including lower depressive symptom scores and higher SRH [31]. Second, a greater relative proportion of SB was associated with higher levels of depressive symptoms and poorer perceived health [32, 33]. Third, LPA showed limited and generally non‐significant associations with the outcomes considered. The isotemporal substitution analyses further supported these patterns. Reallocating time toward MVPA, particularly from SB, was associated with lower predicted depressive symptom scores and higher SRH [34, 35]. Conversely, reallocations away from MVPA toward SB or LPA were associated with less favourable predicted outcomes. In contrast, substitutions between LPA and SB were associated with small and mostly non‐significant changes in the outcomes examined. These findings suggest that, within the daily behavioural composition, MVPA appears to play a particularly relevant role in relation to mental health and perceived health among university students [36, 37]. Although the magnitude of the associations was modest, the direction of the effects was consistent across analytical approaches, including correlation analyses, compositional regression models and isotemporal substitution modelling. The consistency of these findings reinforces the importance of considering movement behaviours as a finite and interdependent composition of time‐use behaviours rather than as independent exposures. The association observed for SRH should also be considered within a broader lifestyle framework. In the same Italian university setting, healthier dietary patterns, particularly greater adherence to the Mediterranean diet, have been associated with better SRH, suggesting that perceived health in young adults reflects multiple interrelated behavioural domains [38, 39].

With respect to ED‐related symptomatology, the observed associations were smaller compared with those identified for depressive symptoms and SRH. Nevertheless, the models suggested that reallocating time from SB toward MVPA was associated with a modest reduction in SCOFF scores, indicating that movement behaviour composition may also relate, albeit more weakly, to ED‐related symptomatology. This finding should be interpreted within the broader clustering of ED‐related symptomatology with mental health and lifestyle factors. In Italian university students, clinically relevant depressive symptoms and poorer SRH have shown particularly strong associations with SCOFF‐defined symptomatology, together with BMI and other behavioural factors [40].

### 4.1. Potential Biological Mechanisms

The observed associations between movement behaviours and mental health outcomes may be underpinned by several biological mechanisms. MVPA has been consistently linked to neurobiological processes that support mental well‐being [41], including increased release of endorphins and monoamines (e.g., serotonin and dopamine) [42], which play a key role in mood regulation. Additionally, MVPA promotes neuroplasticity through upregulation of brain‐derived neurotrophic factor (BDNF) [43, 44], facilitating hippocampal function and potentially reducing depressive symptoms [45, 46]. Regular physical activity is also associated with reduced systemic inflammation and improved hypothalamic–pituitary–adrenal (HPA) axis regulation, both of which are implicated in depression [47]. In contrast, prolonged SB may contribute to adverse mental health outcomes through metabolic dysregulation, increased inflammatory markers and reduced cerebral blood flow, which can negatively affect cognitive and emotional processes [48]. The lack of strong associations for LPA may reflect its lower physiological intensity, which might be insufficient to trigger substantial neurobiological adaptations [49]. Together, these mechanisms provide a plausible biological basis for the beneficial effects of reallocating time toward MVPA and away from SBs observed in this study.

### 4.2. Comparison With International Evidence

Our findings are broadly consistent with the evidence from university populations in other geographical settings. Among Chinese university students, an accelerometer‐based isotemporal substitution analysis showed that replacing 30 min of SB with MVPA was associated with lower depressive and anxiety symptom scores, while replacement with LPA was also associated with lower depressive symptoms [50]. Similarly, in a large multicentre study including more than 8000 undergraduate students from eight Brazilian public universities, reallocating sedentary time to moderate‐ or vigorous‐intensity physical activity was associated with lower odds of depressive and anxiety symptoms [51]. These findings are consistent with the direction of the associations observed in our Italian sample and suggest that replacing sedentary time with more active behaviours, particularly higher‐intensity activity, may be relevant across different university settings. However, unlike the Chinese study, we found limited associations for LPA. Differences in movement‐behaviour assessment, behavioural context and characteristics of the student populations may partly explain this discrepancy.

### 4.3. Public Health Implications

These findings highlight the relevance of MVPA as a key target for improving mental and perceived health among university students.

Even small reallocations of time (e.g., 15 min) from SB to MVPA were associated with modest improvements in depressive symptoms and SRH. Although the magnitude of these associations was small, such time reallocations may represent feasible behavioural targets. Their clinical and public health relevance should, however, be confirmed in longitudinal and intervention studies.

Universities represent a strategic setting for early preventive interventions, given the high burden of psychological distress in this population. Health promotion strategies may therefore encourage both increasing MVPA and reducing sedentary time, while adopting a compositional perspective that considers the interdependence of daily movement behaviours.

Intervention evidence provides some support for translating these findings into university‐based health promotion strategies. A recent systematic review and meta‐analysis including 59 studies found that physical activity interventions in undergraduate students were associated with reductions in depressive, anxiety and stress symptoms, although the certainty of evidence was low and substantial heterogeneity across interventions was observed [52]. Digital strategies may represent an additional, scalable approach: e‐health interventions have been specifically evaluated to promote physical activity and reduce SB among college students [53]. Moreover, a randomised trial conducted among US college students showed reductions in depressive symptoms following an 8‐week web‐based programme delivering either aerobic‐resistance exercise or yoga‐mindfulness sessions [54]. In the Italian university context, these findings support the evaluation of flexible strategies combining accessible opportunities for structured physical activity with digitally supported programmes, rather than relying exclusively on generic recommendations to increase activity.

These results also support a shift toward integrated 24‐h movement approaches, emphasising time reallocation (i.e., ‘move more and sit less’) rather than isolated behavioural targets. Although associations with ED‐related symptomatology were smaller, the findings suggest a potential broader role of movement behaviours in mental health, reinforcing the value of integrated lifestyle interventions in young adults.

### 4.4. Strengths and Limitations

This study has several strengths. First, it includes a large sample of university students (*n* > 2600), enhancing statistical power and allowing for robust estimation of associations. Second, the application of isotemporal substitution modelling allows for the translation of findings into actionable public health messages by quantifying the expected impact of reallocating time between behaviours. Additionally, the use of validated instruments for key outcomes (PHQ‐9, SCOFF and SRH) strengthens the reliability and comparability of the findings.

However, several limitations should be acknowledged. The cross‐sectional design precludes causal inference, and reverse causality cannot be excluded (e.g., individuals with poorer mental health may engage in less physical activity). Movement behaviours were assessed through self‐reported measures, which are subject to recall and social desirability bias, potentially leading to misclassification. The study sample, although large, was drawn from a single university, which may limit generalizability to other populations or cultural contexts. Furthermore, residual confounding cannot be ruled out, as other relevant factors (e.g., diet, sleep quality, socioeconomic status or mental health history) were not included in the models. The modest effect sizes also indicate that movement behaviours represent only one component within a broader multifactorial framework influencing health and well‐being. Indeed, recent evidence suggests that population health outcomes are shaped by interacting behavioural, socioeconomic, and structural determinants, while lifestyle behaviours may cluster with broader conditions of social vulnerability and disadvantage [55, 56]. Lastly, due to technical issues in the Microsoft form, approximately 30 participants have been removed because of missing data.

### 4.5. Future Directions

Future research should prioritise longitudinal and experimental designs to clarify the directionality and causal nature of the associations observed. There is also a need to integrate additional behavioural domains, such as sleep and dietary patterns, within a 24‐h compositional framework to better capture the complexity of lifestyle behaviours and their combined effects on health. The inclusion of objective measures (e.g., accelerometry) would further strengthen the accuracy of movement behaviour assessment.

Finally, translating these findings into real‐world settings will require the development and evaluation of scalable, context‐specific interventions within universities. Future studies in Italian university populations should test pragmatic multicomponent strategies combining structured opportunities for MVPA, approaches to interrupt or reduce prolonged sedentary time, and digitally supported programmes. Multicentre longitudinal studies and randomised trials across different Italian universities would also help determine whether the relatively small associations observed in the present study are reproducible across academic settings and translate into clinically and practically relevant changes in mental health.

## 5. Perspective

In this study, a higher relative allocation of time to MVPA was consistently associated with more favourable mental and perceived health profiles, whereas SB showed the opposite pattern. These findings underscore the importance of considering movement behaviours as a compositional construct and support the potential association between reallocating time with more active behaviours in young adults.

## Author Contributions

**Flavia Pennisi:** writing – original draft, software, methodology, formal analysis, data curation, conceptualisation. **Antonio Pinto:** writing – original draft, visualisation, methodology, data curation, conceptualisation. **Marco Colizzi and Andrea Cozza**: writing – review and editing. **Carlo Signorelli and Vincenzo Baldo**: writing – review and editing, supervision. **Vincenza Gianfredi:** conceptualisation, writing – original draft, software, methodology, investigation, formal analysis, data curation, writing – review and editing, supervision.

## Funding

This research was funded by the Department of Biomedical Sciences for Health, University of Milan (Grant PSR‐LINEA2 2022). Open access publishing facilitated by Universita degli Studi di Padova, as part of the Wiley ‐ CRUI‐CARE agreement.

## Conflicts of Interest

Marco Colizzi reports honoraria from Janssen‐Cilag S.p.A. and Epitech Group S.p.A. for scientific communication and outreach activities and has served as a consultant/advisor for GW Pharma Limited, F. Hoffmann‐La Roche Limited, GW Pharma Italy S.r.l., Idorsia Pharmaceuticals Italy S.r.l. and Insights Driven Research (IDR). Vincenza Gianfredi reports financial support was provided by University of Milan. The other authors declare no conflicts of interest.

## Supporting Information

Additional supporting information can be found online in the Supporting Information section.

## Supporting information


**Supporting Information** The Supporting Information includes Table S1, which provides the completed STROBE Statement checklist for cross‐sectional studies.

## Data Availability

The datasets generated and/or analysed during the current study are available from the corresponding author upon reasonable request.
